# Returnee Executives, Corporate Social Responsibility, and Stock Price Synchronicity

**DOI:** 10.3389/fpsyg.2022.950436

**Published:** 2022-07-13

**Authors:** Di Gao, Yuan Zhao, Qinghua Tian

**Affiliations:** School of International Business, Southwestern University of Finance and Economics, Chengdu, China

**Keywords:** corporate social responsibility, stock price synchronicity, corporate sustainable development, returnee executives, information asymmetry

## Abstract

Executive characteristics have a significant impact on corporate decision-making, corporate sustainable behavior, and stock market performance, which may influence the corporations’ sustainable development in the long run. The role of returnee talents in the corporate sustainable development has received extensive academic attention. Using data of Chinese A-share listed companies over the period of 2008–2018, we find that there is a negative relationship between executives’ foreign experience and stock price synchronicity. We also prove that corporate social responsibility (CSR) has a significant mediating effect on the relationship between returnee executives and stock price synchronicity. The returnee executives tend to pursue long-term sustainable activities and improve CSR engagement quality, thereby reducing stock price synchronicity. Our extended analysis reveals that the benefit of returnee executives is more pronounced for non-SOEs and for firms located in regions with a low degree of marketization. This study has some implications for the Chinese firms in relation to their CSR information disclosure behavior, and it gives suggestions to strengthen capital market efficiency for the sustainable development of corporations.

## Introduction

The executives, as the decision-maker and implementer of corporate strategy, play an essential role in corporate sustainable development. Prior researchers investigate the economic consequences of executives’ personal experience and background from the perspective of academic experience (White et al., 2014), military experience (Benmelech and Frydman, 2015), pilot experience (Cain and McKeon, 2016), acquisition experience (Field and Mkrtchyan, 2017), and early-life experience (Bernile et al., 2017). Recently, executives’ foreign experience has attracted more academic attention. For example, Giannetti et al. (2015) investigate the impact of directors with foreign experience on firm performance in China. Yuan and Wen (2018) attempt to explain the channels by which foreign experience could promote firm performance and they find that managers with foreign experience not only contribute to R&D investment, but also contribute to the increase of patents. With the development of global market integration and international talent communication, the number of returnee executives from overseas increases rapidly. From 1978 to 2018, among the five million people who had studied in abroad and the total number of people who had completed their studies totaled more than 4 million, 84.46% of them chose to return to China. Generally, returnee executives are believed to be conducive to corporate sustainable development in China. By 2020, the number of overseas returnees sending resumes to domestic posts increased by 33.9% from 2019, and the growth rate is much higher than that of 2019 (5.3%) and 2018 (4.3%).

The overseas talents not only have impact on firm decision-making, but also can affect stock market information transmission. According to the prior literature, a returnee executive can help to improve the efficiency of corporate sustainable development through international expansion, innovation, corporate governance, and corporate social responsibility (CSR) strategies at the firm level (Giannetti et al., 2015; Iliev and Roth, 2018; Yuan and Wen, 2018; Zhang et al., 2018; Conyon et al., 2019). In addition, Cao et al. (2018) conduct empirical research based on the data of Chinese listed firms and show the evidence that the uncertainty of directors’ foreign experience is negatively related to crash risk. Returnee executives tend to be more likely to enhance the timeliness of financial reporting (Dobija and Puawska, 2021). Meanwhile, a growing body of the literature provides evidence that excessive stock price synchronicity can seriously damage the resource allocation function of the capital market, and even leading to financing difficulties, hindering enterprises’ sustainable development (Wurgler, 2000; Gul et al., 2011; Morck et al., 2013). Stock price synchronicity, a measure of the degree to which individual stocks co-move with the market, indicates information transmission efficiency in capital market. In comparison with the developed markets, the cost of acquiring private information is much higher in emerging markets and the profitability of informed trading is lower, which leads to higher stock price synchronicity (Gul et al., 2010). As China’s capital market is at the early stage of development and the regulations on corporate information disclosure are not fully-forced, listed firms have the incentives to selectively disclose private information to outside investors. The stock price synchronicity in China is always much higher than that in the developed markets (Morck et al., 2000; Jin and Myers, 2006). However, whether returnee executives can affect stock price synchronicity is still unclear. Thus, this paper reveals the role of returnee executives in explaining the synchronicity of stock price movements and investigates the significance of corporate sustainable behaviors.

Corporate social responsibility (CSR) is an important long-term investment for a firm; it is a firm’s sustainable development behavior as well as an important issue of information transmission in stock markets. According to Gelb and Strawser (2001), firms with high CSR are more likely to provide informative disclosures. Executives’ foreign experience may help to improve the informativeness of stock prices by improving corporate social responsibility strategy, thereby disseminating a firm’s specific information to investors. On the one hand, firms with returnee talents are more likely to enhance their corporate governance, thus improving firms’ foreign acquisition performance and executives’ incentive mechanism (Giannetti et al., 2015; Conyon et al., 2019). On the other hand, agency theory shows that self-interested managers may hide bad news and have short-decision-horizon problems, which influence firms’ long-term success, and thereby, agency cost may arise (Antia et al., 2010; Kim et al., 2014). Since executives with foreign experience have a broader vision and reduced managerial myopia, they may influence firms’ CSR-related decision-making. Therefore, executives with foreign experience are more likely to pursue long-term investment activities. They have the intention to improve CSR engagement quality (Zhang et al., 2018). According to the above statements, we posit that firms with returnee executives are likely to have reliable and high-quality information available to the public, and their stock prices should be less synchronous with the market, which results in the lower stock price synchronicity.

Using data from the China Stock Market & Accounting Research (CSMAR) over the period of 2008–2018, we examine whether executives’ foreign experience is related to stock price synchronicity. We also explore the potential channels through which returnee executives affect the stock market by analyzing the mediating effect of CSR. The results show that returnee executives can significantly reduce stock price synchronicity. The main findings withstand checks for endogeneity, self-selection bias, and robustness tests. Additionally, the mediating analysis indicates that returnee executives reduce stock price synchronicity through CSR engagement. Further analyses indicate that executives’ foreign working experience and foreign studying experience have important impacts on stock price synchronicity. For the non-state-owned enterprises (non-SOEs) and the firms located in the low-marketization regions, executives with foreign experience have a more significant impact on stock price synchronicity.

Our manuscript makes three major contributions to the literature. First, this study examines the role of returnee executives in reducing stock price synchronicity and enriches the existing literature on the influence of executives’ background information. Previous literature has shown the influence of executives’ experience on international exposure (Lee and Park, 2008; Iliev and Roth, 2018), innovation (Yuan and Wen, 2018), firm performance (Giannetti et al., 2015), CSR strategy (Zhang et al., 2018), financial reporting (Dobija and Puawska, 2021), and stock crash risk (Cao et al., 2018), but less is known about the impact of returnee executives on stock price synchronicity. We provide evidence that executives’ foreign experience has a negative impact on stock price synchronicity. Second, this manuscript explores the mechanism by analyzing the mediating effect of CSR, and we prove that executives’ foreign experience can significantly affect CSR practices. Given the significant impact of executives’ foreign experience on corporate success, our study highlights the importance of executives’ foreign experience in corporate information transmission and corporate sustainable development in emerging markets.

The remainder of this manuscript is organized as follows. Section “literature background and hypotheses development” reviews the literature and develops the hypotheses. Section “Research Design” describes data, variables, and model design. Section “Empirical Results and Analysis” presents the empirical results, the robustness checks, and heterogeneity analysis. Section “Conclusion” concludes the manuscript.

## Literature Review and Hypotheses Development

### Returnee Executives and Corporate Sustainable Behavior

Exploring the influencing factors of corporate sustainable behavior from the executives’ characteristics is a hot topic in the recent years. According to the upper echelons theory (Hambrick and Mason, 1984), the personality characteristics and cognitive model of executives will affect the strategic decision-making choices. Thus, executives have a statistically significant effect on corporate behavior and to achieve sustainable competitive advantage (White et al., 2014; Benmelech and Frydman, 2015; Bernile et al., 2017; Field and Mkrtchyan, 2017). Internationalization exposes executives to multiplicity of countries with different institutional systems (Xue et al., 2021). Due to the importance of sustainable development for a firm’s global competitiveness, a number of studies have explored executives’ foreign experience that stimulates this corporate behavior. An increasing number of literature has started to empirically study the influence of executives’ foreign experience on the stock market and firm performance (Lee and Park, 2008; Giannetti et al., 2015; Cao et al., 2018; Iliev and Roth, 2018; Yuan and Wen, 2018; Zhang et al., 2018; Conyon et al., 2019; Dobija and Puawska, 2021). For example, Yuan and Wen (2018) attempt to explain the channels by which foreign experience contributes to firm performance and they find that managers with foreign experience not only contribute to R&D investment, but also contribute to the increase of patents. Cao et al. (2018) conduct empirical research based on the data of Chinese listed firms and show the evidence that the uncertainty of directors’ foreign experience is negatively related to crash risk, implying that the imprinting effect and eyeball effect are the underlying mechanisms. Specifically, Zhang et al. (2018) find that returnee directors may influence firms’ CSR engagement by advising managers to adopt additional CSR activities. Corporate sustainable development clearly benefits from returnee executives. However, it is not yet clear how returnee executives affect firms’ stock price synchronicity.

### Returnee Executives and Stock Price Synchronicity

Stock price synchronicity, a measure of the degree to which individual stocks co-move with the market, reflects firm-specific information transmission efficiency in capital market. Once the unique information of firm is not well-reflected by its stock price, the synchronicity would increase (Morck et al., 2000; Durnev et al., 2003). Prior studies investigated the determinants of stock price synchronicity from the perspectives of the information efficiency view. For example, Jin and Myers (2006) extend prior research and find that stock price synchronicity is higher in countries with more opaque information environments, which enables that insiders control firm-specific information flows to the public and therefore reduce information transparency. Wan et al. (2021) state that investors are information-intensive and will adapt their investment strategies to new market patterns. The higher the information transparency, the more information about the firm-specific information that investors could collect, thereby reducing the synchronicity of stock prices. Further studies investigate other determinants of stock price synchronicity, such as ownership concentration (Gul et al., 2010), large controlling shareholders (Boubaker et al., 2014), institutional investors (An and Zhang, 2013), analysts (Jiang et al., 2018), and managerial characteristics (Neifar and Ajili, 2019). In emerging markets, the stock price synchronicity is much higher than that of developed markets (Jin and Myers, 2006). We argue that returnee executives may affect stock market efficiency from the following aspects.

First, returnee executives are more likely to engage in corporate sustainable development, because more private information can be incorporated into the stock price through their activities, thereby improving the information efficiency and reducing stock price synchronicity. As Chinese economy is at the stage of transition, China’s rapid development requires talents urgently, especially those with foreign experience (Yuan and Wen, 2018; Zhang et al., 2018). Foreign experience refers to the particular experience of the individuals; the returnee executives consider it as a crucial part of the accumulation of human and social capital, including international knowledge and valuable foreign networks (Conyon et al., 2019). The executives with foreign experience could affect the decision-making process of the enterprises, and they are expected to be good at tackling the international challenge faced by the firms (e.g., Piaskowska and Trojanowski, 2014; Iliev and Roth, 2018). It is therefore vital and urgent to discuss the influence of returnee executives on firm-level decisions and capital market allocation (Cao et al., 2018). Second, the returnees may benefit firms initiatively to promote corporate governance and improve the quality of information disclosure. Prior studies find that specialized foreign expertise and networks contribute to corporate innovation, corporate governance, firm performance, and executives’ incentive mechanism (Giannetti et al., 2015; Yuan and Wen, 2018; Conyon et al., 2019). In addition, returnee executives tend to be more likely to enhance the timeliness of financial reporting (Dobija and Puawska, 2021). Stock price synchronicity is affected by information opacity (Jin and Myers, 2006), and better corporate governance should reduce stock price synchronicity by mitigating the risk caused by information asymmetry (Ashbaugh et al., 2006). Thus, in comparison with firms without returnee executive, firms with the returnee talents are more likely to enhance the corporate sustainable development, and as a result choose to disclose more firms’ unique information and reduce stock price synchronicity.

In summary, we posit that firms with returnee executives are likely to have more reliable and high-quality information available to the public, which results in lower stock price synchronicity.

**Hypothesis 1.** There is negative association between total stock price synchronicity and returnee executives.

### Returnee Executives, Corporate Social Responsibility, and Stock Price Synchronicity

As an important long-term investment and a sustainable development behavior for a firm, CSR significantly affects influence stock price synchronicity. A large number of studies examine the determinants of CSR, as well as the economic consequences of CSR. Gelb and Strawser (2001) show evidence that socially responsible firms provide more financial disclosure. Kim et al. (2012) show that socially responsible firms exhibit less evidence of both accrual-based and real-activity earnings’ management. A number of empirical studies find evidence suggesting that CSR is associated with transparent and reliable financial information, and it has positive impact on other dimensions of firm sustainable behavior and outcomes (Lee and Faff, 2009; Kim et al., 2012). In addition, Kim et al. (2014) argue that socially responsible firms commit to a high standard of information transparency and engage in less bad news hoarding, and they prove that firms’ CSR performance is negatively associated with stock price crash risk. A study of Kent and Bu (2020) indicates that investors recognize their firm-specific information and adjust investment behavior when companies report the indirect method.

From the agency cost perspective, managers have an incentive to cover up their self-serving behaviors regardless of shareholders’ interests, by withholding unfavorable information or selectively disclosing information or opportunistically timing the release of value relevant, private information to the market (Jensen and Meckling, 1976; Antia et al., 2010; Kim et al., 2014). Thus, self-interested managers deter the flow of firm-specific information to the market, which contributes to more opaque information environments. As the core figures of enterprise operation and the executors to achieve the objectives of the board of directors, executives with foreign experience are more likely to have a broader vision and reduced managerial myopia. Zhang et al. (2018) prove returnee directors improve firms’ CSR engagement by advising managers to adopt additional CSR activities in China. They state that firms integrate not only the knowledge and skills of returnee directors but also their stakeholder-oriented business approach. In that case, better CSR engagement can encourage investors to collect and trade on proprietary information. Besides, executives with foreign experience are more likely to pursue long-term investment success and promote financial reporting quality (Dobija and Puawska, 2021).

This role of CSR is particularly important in Chinese stock market. Since CSR engagement plays an important role in reducing stock price synchronicity, we predict that firms with returnee executives can alleviate agency conflicts and enhance firms’ CSR engagement efficiently, which is helpful to decrease stock price synchronicity. Our second hypothesis is formalized as follows:

**Hypothesis 2:** CSR plays a mediating role in transmitting the impact of returnee executives on stock price synchronicity.

## Research Design

### Data

In this study, the sample of data is collected on Chinese A-share listed firms during the period 2008–2018. All financial data and data about CSR and executives’ background information were retrieved from the China Stock Market and Accounting Research (CSMAR) database. We choose 2008 as the beginning year of the sample period because the overseas background data of senior executives disclose normally from 2008. Samples were screened according to the following criteria: (1) we excluded financial firms and insurance firm from the sample, (2) we excluded listed firms subjected to special treatment (ST and ST*), (3) we excluded firms with annual trading weeks less than 30 weeks, and (4) the variables were winsorized at both the top and bottom of 1% quantiles to reduce the potential impact of outliers. Following these procedures, the final sample comprised of 23,805 firm-year observations.

### Dependent Variable

The dependent variable is stock price synchronicity. The stock price synchronicity can be used as a proxy for information efficiency in the capital market (Morck et al., 2000). According to the Efficient Markets Hypothesis (EMH), when the information efficiency is relatively high, more information with company traits will be contained in the stock price, which makes it hard to fluctuate with market, thereby showing low stock price synchronicity. Following Morck et al. (2000), Jin and Myers (2006), and Boubaker et al. (2014), we estimate stock price synchronicity for each firm in a particular year using *R*^2^ from the following market model:


(1) $$R\,E\,T_{i,w}=\alpha +\beta_{1}\,M\,K\,T\,R\,E\,T_{i,w}+\beta_{2}\,M\,K\,T\,R\,E\,T_{i,w-1}+\beta_{3}\,I\,N\,D\,R\,E\,T_{i,w}+\beta_{3}\,I\,N\,D\,R\,E\,T_{i,w-1}+\varepsilon_{i.w}$$

where _*RET_i,w*_ is firm *i*’s return on week *w*, _*MKTRET_i,w*_ is the value weighted market return for week w, and _*NDRET_i,w*_ is the industry value-weighted return excluding firm i’s weekly return. _ε_i.w_ represents unspecified random factors.

Synchronicity is often measured by the regression’s *R*-squared value of individual stock returns on market and industry indexes. The larger *R*-squared an individual firm has, the more its stock prices are synchronous with market and/or industry returns. The *R*-squared value is obtained from the above regression. As the value of *R*^2^ is bounded by zero and one, we need to apply a logistic transformation of the *R*^2^ in the empirical analyses to make *R*^2^ meet the normal distribution. Therefore, our synchronicity proxy is defined as follows:


(2) $$S\,Y\,N\,C\,H_{i}=\log\left(\frac{R_{t}^{2}}{1-R_{t}^{2}}\right)$$

where _*R_t ^2*_ is the *R*-squared value from Equation 1 for firm *i* in year *t* and _*SYNCH_i*_ is our empirical measure of annual synchronicity for firm *i*. We also follow Durnev et al. (2003) and Gul et al. (2010) to calculate the fitting coefficient *R*^2^ and use other measures to proxy the stock price synchronicity in the robustness test.

### Independent Variable

The independent variable is executives with foreign experience. Following prior studies (e.g., Yuan and Wen, 2018; Zhang et al., 2018; Conyon et al., 2019), we employ two measures. The first measure, *Foreign_N*, is the number of executives with foreign experience in a company in a given year. The second, *Foreign_D*, counts for 1 if there is at least one executive with foreign experience, and 0 otherwise.

### Mediating Variable

Corporate social responsibility (CSR) is the mediating variable. It is measured by the CSR score of the listed firm that mainly obtained from the Running’s CSR report. Following Zhang et al. (2018), we collect CSR score for Chinese listed firms and use this quantitative indicator to measure the quality of CSR strategy.

### Control Variables

The control variables are firm characteristics which are typically used as the determinants of stock price synchronicity (e.g., Gul et al., 2010; Boubaker et al., 2014). The control variables include firm size (*Size*, the natural logarithm of total assets), return on assets (*ROA*, net profit scaled by total assets), leverage (*Lev*, liabilities scaled by total assets), growth opportunity (*Growth*, annual growth rate of sales), proportion of fixed assets (*FIX*, fixes asset scaled by total asset), market value to book value ratio (*MB*, total market value of the company’s stock divided by the book value of the net assets), and stock turnover rate (*TurnR*, the number of shares traded in the current year divided by the number of shares outstanding). Appendix Table A1 provides the definitions of all variables used in our analysis.

### Model Design

To investigate the effect of executives’ foreign experience on synchronicity (H1), we estimate the following regression model:


(3) $$S\,Y\,N_{i,t}=\alpha +\beta_{1}\,F\,o\,r\,e\,i\,g\,n_{i,t}+\sum C\,o\,n\,t\,r\,o\,l+I\,n\,d\,u\,s\,t\,r\,y+Y\,e\,a\,r+\varepsilon_{i.t}$$

where for firm *i* and year *t*, *Foreign* proxies executives with foreign experience, we employ the two measures *Foreign_N* and *Foreign_D. Control* refers to the set of control variables mentioned above. In addition, the industry and year are included in the model.

To test for the mediating effect of CSR on the relationship between executives’ foreign experience and synchronicity (H2), we estimate the following regression:


(4) $$C\,S\,R_{i,t}=\alpha +\beta_{1}\,F\,o\,r\,e\,i\,g\,n_{i,t}+\sum C\,o\,n\,t\,r\,o\,l+I\,n\,d\,u\,s\,t\,r\,y+Y\,e\,a\,r+\varepsilon_{i.t}$$

(5) $$S\,Y\,N_{i,t}=\alpha +\beta_{1}\,F\,o\,r\,e\,i\,g\,n_{i,t}+\beta_{2}\,C\,S\,R_{i,t}+\sum C\,o\,n\,t\,r\,o\,l+I\,n\,d\,u\,s\,t\,r\,y+Y\,e\,a\,r+\varepsilon_{i.t}$$

where _*CSR_i,t*_ represents the mediating variable. *Control* refers to the control variables, and the effect of industry and year is also controlled in the model.

## Empirical Results and Analysis

### Descriptive Statistics

Table 1 shows the distribution of sample firms by year. The number of executives with foreign experience increases monotonically over the sample period, the proportion of companies with foreign executives has increased from 12% in 2008 to 27% in 2018, reflecting that more and more overseas talents have chosen to return to China and hold important positions in the company in the recent years. The total number of executives with working experience has always been higher than that of executives with learning experience. Although, there is a steady growth of returnee executives in Chinese stock market, the proportion is still relatively low.

**TABLE 1:** Sample distribution.

Year	*N*	*Foreign_N*	*Foreign_D = 1*	*Foreign_E = 1*	*Foreign_W = 1*
2008	1,361	239	158	91	105
2009	1,395	256	176	103	114
2010	1,637	423	266	149	184
2011	1,938	536	343	212	231
2012	2,188	641	432	264	290
2013	2,248	677	469	286	315
2014	2,206	747	496	299	343
2015	2,283	856	558	335	390
2016	2,463	951	633	377	450
2017	2,838	1,193	760	446	559
2018	3,248	1,343	867	466	665
Total	23,805	7,862	5,158	3,028	3,646

Table 2 presents the descriptive statistics for the variables. The mean value of stock price synchronicity (*SYN*) calculated by Equation 1 is −0.322, and the standard deviation is 0.931, which demonstrate that there is a big difference in the stock price synchronicity among sample firms. The mean value of the number of foreign executives in a company (*Foreign_N*) is 0.33, and the mean value of the other dummy variable for foreign executives (*Foreign_D*) is 0.217, indicating that on average, only 21.7% of the companies in the sample have at least one executive with foreign experience. It means that many overseas talents have been attracted by China’s rapid economic development and decided to work in the Chinese listed firms. The mean value of CSR is 38.658, and it ranges from 13.330 to 89.003, indicating that CSR engagement of Chinese listed firms is unbalanced. In the statistical analysis of the control variables, the average firm has a level of total assets (*Size*) of 22.061, and *ROA* has an average (median) of 3.9% (3.7%), whereas leverage (*Lev*) has a mean (median) of 0.436 (0.433). In addition, the firms in our sample have an average fixed asset ratio (*FIX*) of 0.225, an average market value to book value ratio (*MB)* of 0.616, an average sales growth (*Grow*) of 0.441, and an average stock turnover rate (*TurnR*) of 1.656.

**TABLE 2:** Descriptive statistics.

Variable	*N*	Mean	Std Dev	Min	p25	p50	p75	Max
*SYN*	23,805	−0.322	0.931	−3.247	−0.864	−0.227	0.329	1.515
*Foreign_N*	23,805	0.330	0.740	0.000	0.000	0.000	0.000	4.000
*Foreign_D*	23,805	0.217	0.412	0.000	0.000	0.000	0.000	1.000
*CSR*	23,805	38.658	12.319	13.330	30.198	36.014	44.609	89.003
*Size*	23,805	22.061	1.288	19.617	21.121	21.884	22.805	26.022
*ROA*	23,805	0.039	0.054	−0.192	0.014	0.037	0.066	0.192
*Lev*	23,805	0.436	0.210	0.050	0.268	0.433	0.598	0.892
*Grow*	23,805	0.441	1.292	−0.689	−0.036	0.133	0.432	9.631
*FIX*	23,805	0.225	0.168	0.002	0.094	0.190	0.322	0.721
*MB*	23,805	0.616	0.240	0.118	0.430	0.619	0.803	1.112
*TurnR*	23,805	1.656	1.256	0.000	0.749	1.318	2.218	12.863

We use univariate test to show the impact of foreign executives on the stock price synchronicity. Table 3 reports the results. The mean (median) of stock price synchronicity is −0.389 (−0.303) for the firms with foreign experienced executives and −0.303 (−0.209) for the firms without these talents. The differences are statistically significant at the 1% level, which means that firms with foreign experienced executives have lower stock price synchronicity than firms without these talents.

**TABLE 3:** Univariate analysis.

Variable	*Foreign_D = 1*			*Foreign_D = 0*			Differences	
	*N*	Mean	Median	*N*	Mean	Median	Mean Diff.	Median Diff.
*SYN*	5,158	−0.389	−0.303	18,647	−0.303	−0.209	−0.086***	−25.947***
*Size*	5,158	22.204	21.959	18,647	22.021	21.864	0.182***	14.521***
*ROA*	5,158	0.043	0.041	18,647	0.038	0.036	0.004***	45.809***
*Lev*	5,158	0.412	0.409	18,647	0.443	0.439	−0.031***	−40.353***
*Grow*	5,158	0.403	0.145	18,647	0.451	0.130	−0.048**	8.038***
*FIX*	5,158	0.197	0.166	18,647	0.233	0.198	−0.035***	−109.709***
*MB*	5,158	0.603	0.599	18,647	0.619	0.624	−0.017***	−21.653***
*TurnR*	5,158	1.571	1.235	18,647	1.680	1.340	−0.109***	−26.847***

*, **, and *** refer to 10, 5, and 1% levels of significance, respectively.

### Basic Regression Results

#### The Impact of Executives’ Foreign Experience on Stock Price Synchronicity

First, we discuss the influence of executives with foreign experience on stock price synchronicity. The main results of Equation 3 are reported in Table 4. Columns (1) and (2) show that *Foreign_N* and *Foreign_D* are negatively related to *SYN* at the 1% significance level, indicating that executives with foreign experience decrease the stock price synchronicity significantly. Specifically, the coefficient on executives with foreign experience (*Foreign_N*) in Column (3) is −0.040, and the coefficient on *Foreign_D* in Column (4) is −0.062. After controlling year and industry effects, as shown in Columns (3) and (4) of Table 4, executives with foreign experience can significantly reduce stock price synchronicity, both statistically and economically. This verifies Hypothesis 1. For the control variables, the results in Table 4 show that firm performance, leverage, and stock turnover rate are significantly and negatively related to stock price synchronicity, which indicates that firms with better financial performance, more leveraged firms, and firms with higher stock turnover rate could reduce stock price synchronicity. However, firm size is positively and significantly related to stock price synchronicity, suggesting that larger firms have higher stock price synchronicity. The coefficients on fixed asset ratio are found to be insignificant. The coefficients on the control variables are generally consistent with prior studies (Gul et al., 2010; Boubaker et al., 2014; Shen et al., 2021).

**TABLE 4:** The effect of returnee executives on stock price synchronicity.

Variable	*SYN*			
	(1)	(2)	(3)	(4)
*Foreign_N*	−0.053***		−0.040***	
	(−6.57)		(−5.54)	
*Foreign_D*		−0.096***		−0.062***
		(−6.63)		(−4.94)
*Size*	0.093***	0.092***	0.120***	0.119***
	(15.01)	(14.93)	(18.62)	(18.48)
*ROA*	−0.123	−0.126	−0.677***	−0.675***
	(−0.98)	(−1.00)	(−5.89)	(−5.87)
*Lev*	−0.291***	−0.291***	−0.469***	−0.467***
	(−8.06)	(−8.06)	(−14.00)	(−13.94)
*Grow*	−0.015***	−0.016***	−0.024***	−0.024***
	(−3.28)	(−3.31)	(−5.39)	(−5.41)
*FIX*	0.085**	0.081**	−0.006	−0.006
	(2.34)	(2.24)	(−0.15)	(−0.17)
*MB*	0.560***	0.560***	0.486***	0.488***
	(17.47)	(17.48)	(14.89)	(14.96)
*TurnR*	0.034***	0.034***	−0.089***	−0.089***
	(6.34)	(6.34)	(−16.24)	(−16.24)
*Constant*	−2.634***	−2.614***	−1.721***	−1.699***
	(−21.34)	(−21.22)	(−13.33)	(−13.18)
*Year*	No	No	Yes	Yes
*Industry*	No	No	Yes	Yes
*N*	23,805	23,805	23,805	23,805
*R* ^2^	0.044	0.044	0.324	0.323

The t-statistics reported in parentheses are based on standard errors clustered at the firm level. ***, **, and * denote significance at the 1, 5, and 10% levels, respectively.

### Mechanism Analysis

#### The Mediating Effect of Corporate Social Responsibility

Corporate Social Responsibility (CSR), as an important sustainable development behavior for a firm, significantly encourages investors to collect and trade on proprietary information and improve informative disclosure efficiency (Gelb and Strawser, 2001). Therefore, the returnee executives might promote the incorporation of firm-specific information into stock prices, through CSR practices. Further, we examine the mediating role of CSR underlying the effect of executives with foreign experience on stock price synchronicity. As shown in Table 5, we find that the coefficient estimates of *Foreign_N* and *Foreign_D* are negative and significant at the 1% level. Meanwhile, the coefficients on *Foreign_N* and *Foreign_D* are 0039 and 0.066, respectively; both are significant at the 1% level, which indicates that the quality of CSR activities managed by foreign executives is higher. The regression results of Equation 5 are shown in Column 3 of Table 5, and *CSR* is negatively related to *SYN* at the 1% level, suggesting that the higher the quality of CSR engagement, the less stock price synchronicity. Thus, the results support our hypothesis (H2) that returnee executives reduce a company’s stock price synchronicity by improving the quality of CSR engagement. Because of the existence of principal-agent problems, managers have short-decision horizons (Antia et al., 2010). Thus, self-interested managers may make investment decisions at the expense of company prospects and hide bad news from the market for self-interested motivation (Kim et al., 2014). Returnee executives are more likely to reduce managerial myopia, pursue long-term investment activities, and improve governance quality, which leads to improving CSR engagement quality (Zhang et al., 2018).

**TABLE 5:** Mechanism analysis.

Variable	*SYN*	*CSR*	*SYN*	*SYN*	*CSR*	*SYN*
	(1)	(2)	(3)	(4)	(5)	(6)
*Foreign_N*	−0.040***	0.039***	−0.031**			
	(−5.54)	(7.75)	(−2.13)			
*Foreign_D*				−0.062***	0.066***	−0.056*
				(−4.94)	(7.27)	(−1.95)
*CSR*			−0.186***			−0.188***
			(−4.31)			(−4.35)
*Size*	0.120***	0.000	0.120***	0.119***	0.000	0.118***
	(18.62)	(0.74)	(18.55)	(18.48)	(0.59)	(18.40)
*ROA*	−0.677***	−0.054***	−0.698***	−0.675***	−0.054***	−0.459***
	(−5.89)	(−3.17)	(−6.07)	(−5.87)	(−3.16)	(−13.69)
*Lev*	−0.469***	0.020***	−0.461***	−0.467***	0.020***	−0.696***
	(−14.00)	(4.54)	(−13.75)	(−13.94)	(4.60)	(−6.06)
*Grow*	−0.024***	0.004***	−0.023***	−0.024***	0.004***	−0.023***
	(−5.39)	(4.03)	(−5.07)	(−5.41)	(4.03)	(−5.09)
*FIX*	−0.006	−0.055***	−0.019	−0.006	−0.054***	−0.020
	(−0.15)	(−14.59)	(−0.52)	(−0.17)	(−14.51)	(−0.53)
*MB*	0.486***	−0.015***	0.487***	0.488***	−0.014***	0.490***
	(14.89)	(−3.92)	(14.92)	(14.96)	(−3.87)	(14.99)
*TurnR*	−0.089***	0.003***	−0.090***	−0.089***	0.003***	−0.090***
	(−16.24)	(4.62)	(−16.31)	(−16.24)	(4.64)	(−16.31)
*Year*	Yes	Yes	Yes	Yes	Yes	Yes
*Industry*	Yes	Yes	Yes	Yes	Yes	Yes
*Constant*	−1.721***	0.883***	−1.922***	−1.699***	0.858***	−1.904***
	(−13.33)	(10.70)	(−6.84)	(−13.18)	(10.38)	(−6.78)
*N*	23,805	23,805	23,805	23,805	23,805	23,805
*R* ^2^	0.324	0.268	0.085	0.323	0.265	0.085

The t-statistics reported in parentheses are based on standard errors clustered at the firm level. ***, **, and * denote significance at the 1, 5, and 10% levels, respectively.

### Endogeneity Issues and Robustness Test

The relationship between returnee executives and stock price synchronicity investigated in this paper may be affected by other unobservable factors, which may result in endogeneity problems. To address the potential endogeneity issue, we perform difference-in-difference analysis, propensity score matching procedure, Heckman two-step sample selection model, and firm fixed effect model.

#### The Difference-in-Difference Analysis

To alleviate the concern that endogeneity related to omitted variables biases our main finding, we use the exogenous labor supply shock events as a natural experiment to implement the difference-in-difference (DID) analysis. “Since the labor market for board directors is local, the policies to attract highly skilled returnee migrants led to arguably exogenous increases in the supply of potential directors with foreign experience in different provinces at different times,” as argued by Giannetti et al. (2015). The rationale is that, from the late 1990s, a series of provincial governments’ programs aimed at attracting skilled returnees has acted as a form of the so-called human capital supply shock. However, in the recent years, we find that the time of returnee talent introduction policies is different across different cities. By expanding the research depth of exogenous shock (Giannetti et al., 2015), we manually collect the microdata of municipal returnee talent introduction policies, using new city-level regulations for talent returnees as exogenous labor supply shocks, to test whether returnee executives behave differently from non-returnees. We then estimate the following DID regression model:


(6) $$S\,Y\,N_{i,t}=\alpha +\beta_{1}\,T\,r\,e\,a\,t_{i,t}\,\times \,P\,o\,s\,t_{i,t}+\sum C\,o\,n\,t\,r\,o\,l+I\,n\,d\,u\,s\,t\,r\,y+Y\,e\,a\,r+\varepsilon_{i.t}$$

*Treat* is a dummy variable, taking the value of one if a firm that had returnee talents and zero otherwise. *Post* is a dummy variable equal to one in the year that the local government of the listed company promulgates the policy on the introduction of overseas returnees and zero otherwise. Using this specification, we can investigate whether the governments’ programs of attracting skilled returnees are beneficial to reduce the synchronicity of firms with returnees.

Table 6 shows the results of difference-in-difference analysis. Columns (1) and (2) report the results of the full sample. The coefficients on *Treat × Post* are −0.134 and –0.052, respectively, and are significant at 1% level, indicating that the stock price synchronicity of the companies with skilled returnees decreases significantly after the staggered implementation of these regional regulations for talent returnees. The results help us to identify how talent returnees react to exogenous changes in the information environment.

**TABLE 6:** The difference-in difference approach.

Variables	*SYN*	
	(1)	(2)
*Treat × Post*	−0.134***	−0.052***
	(−7.96)	(−3.58)
*Size*	0.093***	0.116***
	(14.63)	(18.30)
*ROA*	−0.136	−0.711***
	(−1.02)	(−6.12)
*Lev*	−0.297***	−0.460***
	(−8.28)	(−13.94)
*Grow*	−0.014***	−0.024***
	(−2.74)	(−5.21)
*FIX*	0.083**	0.003
	(2.28)	(0.07)
*MB*	0.571***	0.487***
	(17.20)	(15.08)
*TurnR*	0.043***	−0.093***
	(7.37)	(−15.98)
*Cons*	−2.646***	−1.642***
	(−21.10)	(−12.91)
*Year*	No	Yes
*Industry*	No	Yes
*N*	23,805	23,805
*R* ^2^	0.045	0.328

The t-statistics reported in parentheses are based on standard errors clustered at the firm level. ***, **, and * denote significance at the 1, 5, and 10% levels, respectively.

#### Propensity Score Matching Procedure

To eliminate this problem and control self-selection bias caused by non-random factors as shown in prior studies, we use propensity score matching procedure following the prior studies (Yuan and Wen, 2018; Conyon et al., 2019). The first step is to predict the selection decision of returnee executives, by estimating a probit model of the binary outcome that equals one if the firm hires an executive with foreign experience, with observable firm characteristics as explanatory variables. Second, we compare firms with foreign-experienced executives (i.e., treatment firms) to a sample of control firms with no foreign-experienced executives (i.e., control firms) matched on the propensity for a firm to appoint executives with foreign experience and then re-estimate model (3) using the treatment and matched control sample.

Table 7 reports the PSM procedure results. The results in Panel A show that firms that hired a returnee executive differ systematically from those that did not, indicating that the selection of executive with foreign experience is strongly endogenous with firm-level characteristics. To ensure that the matching is satisfactory, we assess covariate balance by testing whether the means and medians of the covariates differ between the treatment firms and matched control firms. As Panel B shows, there are no significant differences in the means of any covariates, indicating that the propensity score-matched control sample resembles the treatment firms along virtually all dimensions. At last, we re-estimate model (3) using PSM and report the regression results of our baseline model using the PSM sample. The results remain robust as shown in Panel C.

**TABLE 7:** PSM procedure.

Panel A: First stage of propensity score			
Variable			*Foreign_D*
*Size*			0.161***
			(6.68)
*ROA*			−0.631**
			(−2.07)
*Lev*			−0.499***
			(−4.20)
*Grow*			−0.010
			(−0.75)
*FIX*			−0.697***
			(−4.69)
*MB*			−0.354***
			(−3.35)
*TurnR*			−0.034**
			(−2.30)
*Cons*			−4.075***
			(−8.11)
*Year and Industry*			Yes
*N*			23,805
*Pseudo R* ^2^			0.046

**Panel B: Descriptive statistics for propensity-score matched subsamples**			

**Variable**	***Foreign_D = 1***	***Foreign_D = 0***	***t-stat***

*Size*	22.204	22.207	−0.12
*ROA*	0.043	0.041	1.26
*Lev*	0.412	0.412	0.03
*Grow*	0.403	0.392	0.51
*FIX*	0.197	0.199	−0.47
*MB*	0.603	0.611	−1.78
*TurnR*	1.571	1.553	0.75

**Panel C: Sample matched by propensity score: second stage**		

**Variables**	***SYN***		
	**(1)**	**(2)**

*Foreign_N*	−0.037***	
	(−3.17)	
*Foreign_D*		−0.058***
		(−2.77)
*Size*	0.118***	0.116***
	(7.55)	(7.42)
*ROA*	−1.035***	−1.033***
	(−4.77)	(−4.76)
*Lev*	−0.473***	−0.467***
	(−6.65)	(−6.59)
*Grow*	−0.032***	−0.032***
	(−3.07)	(−3.09)
*FIX*	−0.045	−0.045
	(−0.53)	(−0.53)
*MB*	0.372***	0.376***
	(5.22)	(5.26)
*TurnR*	−0.078***	−0.078***
	(−7.11)	(−7.09)
*Constant*	−1.543***	−1.496***
	(−4.89)	(−4.75)
*Year*	Yes	Yes
*Industry*	Yes	Yes
*N*	10,316	10,316
*R* ^2^	0.296	0.296

The t-statistics reported in parentheses are based on standard errors clustered at the firm level. ***, **, and * denote significance at the 1, 5, and 10% levels, respectively.

#### Heckman Two-Step Estimation

It is possible that the self-selection bias problem remains. A firm’s decision to appoint an executive with foreign experience may be non-random, and it is likely that executives with foreign experience are more likely to be selected by the companies with low stock price synchronicity, which may cause a self-selection bias. In this case, we attempt to alleviate these concerns using the two-stage Heckman (1979) test. In the first step, we estimate a probit model with a binary dummy *Foreign_D* as the dependent variable, which equals 1 if a firm has at least one executive with foreign experience, 0 otherwise. Following the previous literature (Yuan and Wen, 2018), we conduct *Forign_M*, which is the mean of the proportion of returnee executives in other companies in the same industry in the same year. In addition, we add the following determinants of appointing executives with foreign experience: *Size*, *ROA*, *Lev*, *Grow*, *FIX*, *MB*, *TurnR*, and *Foreign_M*. The year and industry effects are controlled. In the second stage, the inverse Mills ratio (IMR) is generated from the first step and then included to control for the potential sample selection bias.

Table 8 reports the regression results of Heckman model. The results of the first-step regression show that *Size* and *Foreign_M* have significant and positive impacts on a firm’s decision to appoint executives with foreign experience. The results of the second-step regressions indicate that after controlling the endogenous problems caused by self-selection bias, returnee executives still significantly reduce stock price synchronicity.

**TABLE 8:** Heckman two-step estimation.

The first stage		The second stage	
*Foreign_D*		*SYN*	
Variable	(1)	(2)	(3)
*Foreign_N*		−0.040***	
		(−5.63)	
*Foreign_D*			−0.064***
			(−5.03)
*IMR*		−0.211**	−0.208**
		(−2.51)	(−2.47)
*Foreign_M*	−10.258***		
	(−6.85)		
*Size*	0.162***	0.094***	0.093***
	(14.41)	(7.67)	(7.60)
*ROA*	−0.621***	−0.576***	−0.576***
	(−3.11)	(−4.74)	(−4.74)
*Lev*	−0.503***	−0.388***	−0.387***
	(−8.37)	(−8.37)	(−8.35)
*Grow*	−0.010	−0.022***	−0.022***
	(−1.24)	(−4.94)	(−4.96)
*FIX*	−0.689***	0.109*	0.107*
	(−9.88)	(1.87)	(1.82)
*MB*	−0.369***	0.542***	0.544***
	(−6.39)	(13.69)	(13.72)
*TurnR*	−0.035***	−0.084***	−0.084***
	(−3.56)	(−14.03)	(−14.03)
*Constant*	−4.079***	−0.888**	−0.878**
	(−17.35)	(−2.49)	(−2.46)
*Year and Industry*	Yes	Yes	Yes
*N*	23,805	23,805	23,805
*Pseudo R* ^2^	0.0485		
*R* ^2^		0.324	0.323

The t-statistics reported in parentheses are based on standard errors clustered at the firm level. ***, **, and * denote significance at the 1, 5, and 10% levels, respectively.

#### Firm Fixed Effect Model

To mitigate potential problems that may arise from omitting time-invariant firm-specific characteristics, we re-estimate the regressions of model (3) using the firm fixed effect model, when *SYN* is adopted as the dependent variable. The regression results of the fixed effect model are shown in Table 9. The results suggest that the estimated coefficients on the variable returnee executives *Foreign_N* and *Foreign_D* are significantly negative at the 1% level. This implies that our results are not driven by time-invariant firm-specific characteristic, and the main findings are robust for endogeneity issues.

**TABLE 9:** Firm fixed effect model.

Variable	*SYN*	
	(1)	(2)
*Foreign_N*	−0.030***	
	(−2.58)	
*Foreign_D*		−0.049**
		(−2.46)
*Size*	0.017	0.017
	(1.20)	(1.19)
*ROA*	−0.560***	−0.559***
	(−4.37)	(−4.36)
*Lev*	−0.096*	−0.095*
	(−1.80)	(−1.78)
*Grow*	−0.022***	−0.022***
	(−4.72)	(−4.72)
*FIX*	0.207***	0.209***
	(3.14)	(3.19)
*MB*	0.685***	0.684***
	(15.89)	(15.85)
*TurnR*	−0.145***	−0.146***
	(−24.51)	(−24.53)
*Constant*	−0.170	−0.166
	(−0.60)	(−0.59)
*Firm*	Yes	Yes
*Year*	Yes	Yes
*N*	23,805	23,805
*R* ^2^	0.332	0.332

The t-statistics reported in parentheses are based on standard errors clustered at the firm level. ***, **, and * denote significance at the 1, 5, and 10% levels, respectively.

### Other Robustness Checks

#### Alternative Methods of Identifying Independent Variables

A total of three alternative approaches are considered to confirm the robustness of our results. First, we use alternative methods of identifying the independent variables of returnee executives. Following Zhang et al. (2018), we use the proportional variable (*Foreign_R*) as a new identifying method for returnee executives. Panel A of Table 10 shows the results. It is observed that the coefficient on *Foreign_R* is still significantly negative at the level of 1%, suggesting that after changing the independent variable identifying method, returnee executives can significantly reduce stock price synchronicity.

**TABLE 10:** Robustness tests.

Panel A: Alternative methods of identifying dependent variables				
Variable				*SYN2*
*Foreign_R*				−0.172***
				(−3.23)
*Control*				Yes
* Year*				Yes
* Industry*				Yes
*Constant*				−1.679***
				(−13.06)
*N*				23,805
*R* ^2^				0.323

**Panel B: Alternative methods of identifying independent variables**				

**Variable**	***SYN2***		***SYN3***	
	**(1)**	**(2)**	**(3)**	**(4)**

*Foreign_N*	−0.028***		−0.048***	
	(−4.63)		(−6.73)	
*Foreign_D*		−0.050***		−0.091***
		(−4.56)		(−7.22)
*Control*	Yes	Yes	Yes	Yes
* Year*	Yes	Yes	Yes	Yes
* Industry*	Yes	Yes	Yes	Yes
*Constant*	−1.198***	−1.186***	−0.442***	−0.425***
	(−10.63)	(−10.54)	(−3.46)	(−3.34)
*N*	23,805	23,805	23,805	23,805
*R* ^2^	0.339	0.339	0.436	0.437

**Panel C: Robustness check: adding control variables**		

**Variable**	***SYN***				
	**(1)**	**(2)**	

*Foreign_N*	−0.034***	
	(−4.66)	
*Foreign_D*		−0.048***	
		(−3.78)	
*Size*	0.106***	0.105***	
	(16.36)	(16.22)	
*ROA*	−0.657***	−0.655***	
	(−5.73)	(−5.71)	
*Lev*	−0.468***	−0.466***	
	(−14.00)	(−13.93)	
*Grow*	−0.023***	−0.023***	
	(−5.14)	(−5.16)	
*FIX*	−0.046	−0.046	
	(−1.26)	(−1.25)	
*MB*	0.502***	0.505***	
	(15.44)	(15.51)	
*TurnR*	−0.090***	−0.090***	
	(−16.30)	(−16.29)	
*Age*	0.014***	0.013***	
	(9.07)	(8.99)	
*Dual*	−0.056***	−0.057***	
	(−4.78)	(−4.87)	
*Tenure*	0.001***	0.001***	
	(3.71)	(3.72)	
* Year*	Yes	Yes	
* Industry*	Yes	Yes	
*Constant*	−2.076***	−2.050***	
	(−15.11)	(−14.95)	
*N*	23,805	23,805	
*R* ^2^	0.327	0.327	

The t-statistics reported in parentheses are based on standard errors clustered at the firm level. ***, **, and * denote significance at the 1, 5, and 10% levels, respectively.

#### Alternative Methods of Identifying Dependent Variables

The regression results of changing the *SYN* calculation method of the dependent variable are shown in Table 10. Following the literature of Piotroski and Roulstone (2004) and Gul et al. (2010), we recalculated *SYN2* and *SYN3*. The regression results prove that the above basic regression results are robust. Panel B of Table 10 presents the results relating to alternative measures of stock price synchronicity. Similarly, the foreign experience variables are significantly and negatively associated with *SYN2* and *SYN3*.

#### Adding Control Variables

Considering that the stock price synchronicity could be affected by the executives’ foreign background as well as other characteristics, such as age, position, tenure, and so on, we add CEO duality, executives’ age, and tenure, and we add more control variables (Krishnan and Parsons, 2008; Gul et al., 2010; Li et al., 2016). The results are tabulated in Panel C of Table 10. As the table shows, *Age* and *Tenure* is positively associated with stock price synchronicity, whereas *Dual* is negatively associated with stock price synchronicity. The relationship between executives’ foreign experience and stock price synchronicity is significantly negative at the 1% level. Overall, the findings provide robust evidence that firms with returnee executives have lower stock price synchronicity.

### Further Analysis

#### Foreign Working Experience vs. Foreign Education Experience

Bernile et al. (2017) find that executives’ different experiences or abilities have significant different impacts on executives’ decision-making style and the signals they send to the capital market. In addition, prior researchers argue that the amount of international education in a management team contributes to corporate innovation and good performance (Yuan and Wen, 2018). Thus, we predict that executives with foreign study experience may have more significant impacts on stock price synchronicity than those with work experience. We construct two variables: *Foreign_E* is a dummy variable, which equals 1 if a firm has at least one executive with foreign education experience. *Foreign_W* is a dummy variable, which equals 1 if a firm has at least one executive with foreign working experience, and 0 otherwise.

We re-estimate model (3) and the regression results are shown in Table 11. From the regression results in Columns (1) and (2), we can see that the regression coefficient on executives with foreign working experience *Foreign_W* and those of foreign education experience *Foreign_E* are significantly at the 1% level, suggesting that both types of experience significantly reduce the stock price synchronicity.

**TABLE 11:** Further analysis.

Variable	*Foreign_W*	*Foreign_E*	*SOEs*	*Non-SOEs*	*MKT_high*	*MKT_low*

	(1)	(2)	(3)	(4)	(5)	(6)
*Foreign_D*	−0.052***	−0.083***	−0.019	−0.032**	−0.021	−0.113***
	(−2.78)	(−3.93)	(−1.44)	(−2.10)	(−0.96)	(−4.06)
*Size*	0.120***	0.114***	0.095***	0.092***	0.027**	0.075***
	(17.20)	(16.13)	(10.35)	(9.46)	(2.27)	(5.94)
*ROA*	−0.751***	−0.599***	−0.156	−0.849***	−0.095	−0.223
	(−6.10)	(−4.79)	(−0.90)	(−5.61)	(−0.44)	(−1.12)
*Lev*	−0.491***	−0.472***	−0.590***	−0.427***	−0.448***	−0.614***
	(−13.81)	(−13.18)	(−11.95)	(−9.18)	(−6.94)	(−9.73)
*Grow*	−0.022***	−0.023***	−0.026***	−0.022***	−0.023**	−0.043***
	(−4.68)	(−4.93)	(−4.37)	(−3.39)	(−2.51)	(−6.40)
*FIX*	0.014	−0.040	−0.230***	0.121**	0.005	−0.154**
	(0.37)	(−1.03)	(−4.68)	(2.17)	(0.07)	(−2.48)
*MB*	0.500***	0.504***	0.591***	0.492***	0.917***	0.704***
	(14.35)	(14.24)	(12.20)	(11.01)	(14.33)	(11.63)
*TurnR*	−0.092***	−0.091***	−0.081***	−0.100***	−0.081***	−0.086***
	(−15.79)	(−15.27)	(−10.02)	(−13.62)	(−8.19)	(−9.31)
* Year*	Yes	Yes	Yes	Yes	Yes	Yes
* Industry*	Yes	Yes	Yes	Yes	Yes	Yes
*Constant*	−1.729***	−1.600***	−1.101***	−1.236***	−0.488*	−0.617**
	(−12.47)	(−11.38)	(−5.96)	(−6.37)	(−1.89)	(−2.47)
*N*	20,777	20,159	9,867	13,938	7,299	6,603
*R* ^2^	0.325	0.323	0.336	0.305	0.332	0.344

The t-statistics reported in parentheses are based on standard errors clustered at the firm level. ***, **, and * denote significance at the 1, 5, and 10% levels, respectively.

#### SOEs vs. Non-SOEs

Considering the actual situation in China, state-owned enterprises are different from non-state-owned enterprises in terms of business operation, management structure, and external environment. Previous studies have documented that SOEs are less efficient than private firms (Dewenter and Malatesta, 2001; Chen et al., 2017). In comparison with non-SOEs, SOEs may not effectively release the content of firm-specific information, and the lack of efficiency may hinder the process, which may lead to higher stock price synchronicity. Consequently, we argue that returnee executives in SOEs are less likely to reduce stock price synchronicity than those in non-SOEs.

We divide the sample into two groups, SOEs and non-SOEs, and employ the regression model (3) to verify whether the impact of returnee executives on the stock price synchronicity will be relatively different in terms of different property rights. The regression results are shown in Columns (3) and (4) of Table 11. This regression results indicate that returnee executives in non-SOEs can play their own role much better in reducing stock price synchronicity and have more incentives to release the content of stock price information than those in SOEs.

#### High Marketization vs. Low Marketization

Property right protection and financial development influence the information efficiency of the capital market by affecting the information collection cost and power of investors. Morck et al. (2000) believe that stock price synchronicity is affected by the strength of the legal system on the protection of property rights. When the property rights of investors cannot be fully guaranteed by law, the information reflecting the value of the company will become invalid. In areas with better legal environment, the implementation of the law inhibits the information occupation of insiders and increases the information content of stock prices, which leads to lower stock price synchronicity (Fernandes and Ferreira, 2009). In that case, returnee executives play a limited role in improving information efficiency, and we predict that the effect of foreign experiences on reducing stock price synchronicity is more significant in regions with of low-degree marketization.

Following Bu et al. (2022), we use a marketization index *MKT* to represent the province-specified marketization degree and divide the sample into two groups. *MKT = high* is a dummy variable, which equals 1 if firm located in regions that the marketization level is higher than median value in a given year, and 0 otherwise. The results of further analysis are presented in Columns (5) and (6) of Table 11. It can be seen that the information transmission requires environment with better financial development and good property rights protection. The coefficient on *Foreign_D* in low degree of marketization subsets is negative and significant at the 5% level. However, the coefficient on *Foreign_D* is insignificant in sample with a high degree of marketization.

## Conclusion

This manuscript examines whether and how executives’ foreign experience and corporate social responsibility affect stock price synchronicity. Using the data of Chinese listed firms from 2008 to 2018, it provides evidence that returnee executives have a significantly negative effect on stock price synchronicity. Through mediating analysis, we find that the negative impact of returnee executives on stock price synchronicity is caused by CSR engagement, which is consistent with the benefits of foreign experience. We also find that the benefit of returnee executives is more pronounced for the non-SOEs or for firms located in regions with a low degree of marketization. Both foreign working experience and foreign education experience of the returnee executives can significantly reduce the stock price synchronicity. Robustness tests support the above results.

Our study emphasizes the importance of returnee executives and CSR practices for corporate sustainability, suggesting that the returnee executives hired by the Chinese listed firms can contribute to corporate information transmission, and they can help Chinese enterprises achieve long-term sustainable development. Based on our findings, we propose the following recommendations. First, firms with knowledgeable and skilled returnee executives can enjoy several advantages; executives’ foreign experience has a positive effect on firms’ CSR strategy and international business integration. The government and enterprises should pay more attention to the executive incentive including executive compensation and policies on talents’ introduction. Reasonable incentive and management mechanism should be established. Second, as the stock price synchronicity in emerging markets is much higher than that in the developed markets, identifying the elements that affect firms’ information transparency has important practical implications. Therefore, to reduce the stock price synchronicity, listed firms should reduce information asymmetry by strengthening corporate governance, CSR practices, and increasing information disclosure quality. At the same time, the relevant regulators are supposed to strengthen the supervision of listed companies, curb the self-interest behavior of managers, and create a good condition for the sustainable development of enterprises. Finally, the sustainable development of enterprises is closely related to the stock price in the capital market. Firms should enhance their CSR engagement and reduce the risk of information asymmetry in their sustainable development, so as to win the trust of their investors in the capital market.

## Data Availability Statement

The original contributions presented in this study are included in the article/supplementary material, further inquiries can be directed to the corresponding author.

## Author Contributions

DG and YZ contributed to the conception and design of the study. QT organized the database. DG and QT performed the statistical analysis. DG wrote the draft of the manuscript. YZ contributed to writing, reviewing, and editing. All authors contributed to manuscript revision, read, and approved the submitted version.

## Conflict of Interest

QT is employed by Shenzhen Branch of China Telecom Corporation Limited. The remaining authors declare that the research was conducted in the absence of any commercial or financial relationships that could be construed as a potential conflict of interest.

## Publisher’s Note

All claims expressed in this article are solely those of the authors and do not necessarily represent those of their affiliated organizations, or those of the publisher, the editors and the reviewers. Any product that may be evaluated in this article, or claim that may be made by its manufacturer, is not guaranteed or endorsed by the publisher.

## Appendix

**Appendix Table A1:** Variable definitions.

Variable	Definition
*SYN*	Logarithmic transformation of *R*^2^ for the market model in Eq. 1, defined as log [*R*^2^/(1 − *R*^2^)], Following Morck et al. (2000); Jin and Myers (2006), and Boubaker et al. (2014) model.
*SYN2, SYN3*	Logarithmic transformation of *R*^2^, defined as log [*R*^2^/(1 − *R*^2^)], following Durnev et al. (2003) and Gul et al. (2010) model
*Foreign_N*	The number of executives with foreign experience
*Foreign_D*	A dummy variable defined as 1 if there is at least one returnee executive and 0 otherwise
*Foreign_W*	A dummy variable defined as 1 if there is at least one executive who has overseas working experience and 0 otherwise (only for further analyses)
*Foreign_E*	A dummy variable defined as 1 if there is at least one executive who has overseas education experience and 0 otherwise (only for further analyses)
*Foreign_R*	A proportional variable defined as the proportion of returnee executives of the total number of executives in the company.
*CSR*	The CSR score of the listed firm obtained from the Running’s CSR report
*Size*	The natural logarithm of total assets
*ROA*	The company’s net profit divided by the total assets
*Lev*	The value of liabilities divided by total assets
*Grow*	The annual growth rate of sales
*FIX*	The sum of fixed assets scaled by total assets
*MB*	The total market value divided by the book value of assets
*TurnR*	The total number of shares traded in a year, divided by the total number of shares outstanding at the end of the fiscal year
*Dual*	A dummy variable defined as 1 if the CEO is also the chairman of the board, and 0 otherwise.
*Age*	The log value of executives’ age.
*Tenure*	The log value of number of years since an executive took office at that company.
